# Genuine “Old Fashioned Gold Foil”

**Published:** 1877-03

**Authors:** 


### GENUINE "OLD FASHIONED GOLD FOIL."

EDITOR DENTAL REGISTER:—I have in my possession forty-eight grains of number six gold foil, manufactured by J. L., of this city, over twenty-nine years ago. Notwithstanding the great improvement in foil during the last quarter of a century, this will pass very well.

I also have a letter from J. L. dated January 22, 1848, in which he says; "There is a new agent for putting folks to sleep that is used here—called chloroform; it is pleasanter to take than the Letheon." A. B.

Cin., Feb. 18, 1877.

